# Effect of Natural Rubber in Polyethylene Composites on Morphology, Mechanical Properties and Biodegradability

**DOI:** 10.3390/polym12020437

**Published:** 2020-02-13

**Authors:** Elena Mastalygina, Ivetta Varyan, Natalya Kolesnikova, Maria Isabel Cabrera Gonzalez, Anatoly Popov

**Affiliations:** 1 Scientific Laboratory “Advanced Composite Materials and Technologies”, Plekhanov Russian University of Economics, 36 Stremyanny per., 117997 Moscow, Russia; 2 Department of Biological and Chemical Physics of Polymers, Emanuel Institute of Biochemical Physics, Russian Academy of Sciences, 4 Kosygina str., 119334 Moscow, Russia; 3 Biología Molecular y Bioquímica, Universidad de Malaga, 2 Cervantes str., 29071 Malaga, Spain

**Keywords:** packaging material, bio-based polymer composite, polyethylene, natural rubber, water absorption, mycological test, biodegradability, mechanical properties

## Abstract

Compounding natural additives with synthetic polymers allows developing more eco-friendly materials with enhanced biodegradability. The composite films based on low-density polyethylene (PE) with different content of natural rubber (NR) (10–30 wt%) were investigated. The influence of NR content on structural features, water absorption and mechanical properties of the composites were studied. The 70PE/30NR composite is characterized by the uniform distribution and the smallest size of NR domains (45 ± 5 μm). A tensile test was satisfied by the mechanical properties of the biocomposites, caused by elasticity of NR domains. The tensile strength of 70PE/30NR composite film is 5 ± 0.25 MPa. Higher water absorption of PE/NR composites (1.5–3.7 wt%) compared to neat PE facilitates penetrating vital activity products of microorganisms. Mycological test with mold fungi and full-scale soil test detected the composite with 30 wt% of NR as the most biodegradable (mass loss was 7.2 wt% for 90 days). According to infrared spectroscopy and differential scanning calorimetry analysis, NR consumption and PE structural changes in the biocomposites after exposure to soil occurred. The PE/NR composites with enhanced biodegradability as well as satisfied mechanical and technological properties have potential applications in packaging and agricultural films.

## 1. Introduction

Synthetic polymers widely used for packaging and agricultural applications are characterized by high resistance to degradation under environmental conditions. As a result, the problem of polymer wastes accumulation has become relevant. One of the most promising approaches for solving this environmental problem is developing biodegradable polymeric materials, including naturally occurring biodegradable polymers, biodegradable polymers derived from renewable resources, biodegradable composites based on petroleum and biodegradable polymers [1].

Among the listed biodegradable polymer materials, the direction of developing biodegradable polymer composites are deemed cost-effective by passing an expensive step of the synthesis [2]. Modification of the synthetic polymer matrix by introducing additives initiating degradation allows obtaining new materials with enhanced biodegradability after the life cycle. In this case, the synthetic polymer being a part of the composite determines the required performance and technological properties, as well as the possibility of recycling. The use of poly(olefins), particularly poly(ethylene), as the polymer matrix for such composites is caused by importance of their utilization and a great deal of wastes based on them [3].

Currently, oxo-additives based on transition metal salts [4,5] are commonly used as degrading additives for poly(olefins). Such additives could initiate the material photo-oxidative degradation by oxygen and sunlight, which is rarely realized in terms of waste disposal.

It is known a series of polyolefin compositions with the addition of naturally occurring biodegradable polymers, such as poly(hydroxyalkanoates) [6,7] or poly(lactic acid) [8,9] required the specific composting conditions. For example, the biodegradability of poly(lactic acid) is fully realized only at an elevated temperature (50–60 °C) [10]. In turn, the materials based on poly(hydroxyalkanoates) have high biodegradability and biocompatibility, but mechanical and physical properties of such materials are too poor [11] that confine their scope of use. Furthermore, the technological difficulties of obtaining polyesters by biosynthesis determine a high cost of the materials based on them. To improve mechanical properties, biopolymer blends transformed into micro- or nanofibrillar biocomposites in which higher melting point biopolymer acts as reinforcement and lower melting point biopolymer acts as a matrix have been developed. For instance, there are studies devoted to microinjection molded poly(lactic acid)-based composites with 10–40 wt% of polybutylene succinate nanofibrils [12], 3–10 wt% of poly(butylene adipate-co-terephthalate), and 20 wt % poly(ε-caprolactone) [13].

The addition of natural fillers to the synthetic polymer matrix allows getting materials with the capability of being decomposed more rapidly under the environmental conditions [14]. The use of natural fillers derived from renewable resources allows, on the one hand, to replace non-renewable petrochemical raw materials with renewable those; on the other hand, to reduce the production cost by using manufacturing and agricultural waste products. The numerous research achievements have been devoted to the composites based on low-density or high-density poly(ethylene) filled with cellulose [15] and cellulosic materials such as wood flour, flax shive [16], rice husk [17], corn [18], sisal and hemp [19] fibers, as well as banana flour [20] and soy protein [21]. The high ability of natural polymers to be assimilated by microorganisms determines the intensification of biofouling on the hybrid composite polymeric materials. Unfortunately, the main weakness of such materials is related to lower mechanical properties compared with traditional polyolefins.

The additive of natural rubber appears to have a great potential for using as a component initiating the biodegradation of poly(ethylene) composites. Natural rubber had been reported to be susceptible to biodegradation by a wide range of bacteria [22] and different cultures of mold fungi [23]. The main component of natural rubber is poly(isoprene). According to the previous studies, poly(isoprene) component forms flexible nano-scale or micro-scale drops in the matrix of poly(ethylene) [24]. The size of elastomeric drops in the poly(ethylene) matrix depends on natural rubber content and blending technology. Due to its low modulus of elasticity and low tensile strength, elastomeric natural rubber cannot act as reinforcing filler for poly(ethylene). In this study, the investigation of structure, physical-mechanical properties and biodegradability of the composites based on low-density poly(ethylene) with natural rubber was carried out. According to the previous results [25], the significant modification of the poly(ethylene) crystalline phase in the poly(ethylene)/natural rubber composites affected by moisture and environmental factors have been determined. This paper is focused mostly on investigating of thermophysical and mechanical properties of the composite films based on poly(ethylene) and natural rubber. The biodegradability of the materials under investigation was analyzed by a microbiological test with mold fungi and a full-scale test in the soil medium.

## 2. Materials and Methods 

### 2.1. Materials

The objects of the study were low-density poly(ethylene) (PE, grade 15803-020 from Neftekhimsevilen, OJSC, Kazan, Russia), natural rubber (NR, grade SVR 3L, Dong Xoai, Vietnam), synthetic isoprene rubber (SRI, grade SRI-3, Sintez Kauchuk, OJSC, Sterlitamak, Russia) and double composites of poly(ethylene)/natural rubber (PE/NR) containing 10, 20, 30 wt% of NR. The characteristics of low-density poly(ethylene) used in this study are given in Table 1 [15].

The natural rubber under investigation consists of poly(cis-1,4-isoprene), with minor impurities of other compounds and water (Table 2) [26].

All the blends for this study were prepared by a Brabender mixing system (ICHP, Moscow, Russia) in an argon atmosphere (State Standard GOST 10157-79) at a temperature of (140 ± 2) °C and a rotor rotational speed of 30 rev·min^−1^. Before processing the composites, natural rubber was granulated. The initial size of NR pellets was 3–5 mm. A weighted amount of PE was placed in the mixing chamber, and 2 minutes later, NR was added. Mixing of polymers was performed for 5 min. An inert argon atmosphere was used to reduce the oxidation of polymers. After cooling, the samples were milled using a knife mill RM-120 (Vibrotechnik, LLC, St. Petersburg, Russia) and then compressed by a manual hydraulic press PRG-10 (VNIR, LLC, Moscow, Russia) at a temperature of (190 ± 2) °C and under a load of 0.78 kN on a cellophane substrate followed by quenching in water at (20 ± 2) °C. As a result, the film round samples with a diameter of 8 cm and a thickness of (120 ± 10) µm were obtained.

### 2.2. Methods

#### 2.2.1. Optical Microscopy

The NR domain size and the uniformity of NR particle distribution in the PE matrix were investigated under an optical microscope Axio Imager Z2m (Carl Zeiss, Oberkochen, Germany) in the transmitted and reflected light (at a magnification of 100× and 200×). The average value of domains’ diameter (D) was obtained through measurements of 10 domains in the 10 microscopic fields. Based on the conducted analysis, the size distribution of NR domains in the composites with different content of NR was obtained. The intensity of microorganisms’ growth was investigated in the transmitted light (at a magnification of 100×).

#### 2.2.2. Mechanical Properties (Tensile Test)

The mechanical properties were studied using a tensile machine GP UG 5 DLC-0,5 (DVT Devotrans, Istanbul, Turkey) at a rate of 100 mm/min in accordance with BS EN ISO 527-1 and BS EN ISO 527-3 (7 measurements for each point). Test samples corresponded to 1B type (EN ISO 527-3, the working length was 40 mm) were prepared from the films by a punching press. The number of samples of each composition was 7. The extreme values were excluded from the results. The modulus of elasticity (Young’s modulus) was determined by the stress-strain curves in the field of elastic deformation (the interval between two strains was from 0.1% to 0.3%).

#### 2.2.3. Differential Scanning Calorimetry (DSC)

The behavior of PE in the composites during melting and crystallization was studied using a differential scanning calorimeter DSM-10M (IBI, Pushchino, Russia) in a temperature range from 40 to 150 °C at a scanning rate of 8 °C·min^−1^. The sample weight was (10 ± 0.1) mg. The temperature scale was calibrated by an indium standard (*T_m_* = 156.6 °C, Δ*H* = 28.44 J·g^−1^). To obtain cooling curves, the samples of PE/NR blends were heated up to 150 °C, stored at this temperature for 5 min, then cooled at a rate of 8 °C·min^−1^ to 40 °C. The temperatures of melting and crystallization (*T_m_* and *T_c_*) were determined by an endothermic maximum of the melting peak and an exothermic maximum of the crystallization peak on DSC thermograms, respectively. The enthalpy of fusion (∆*H_i_*) was calculated from the area of a melting peak limited by the baseline. Each value of the parameters Δ*H_i_*, *T_m_*, *T_c_* was obtained by averaging of 5 measurements. To calculate the degree of PE crystallinity (*χ*), Equation (1) was used [27], where Δ*H_i_* is the specific heat of melting calculated per the content of PE in the composite, Δ*H_o_* = 293 J·g^−1^—the specific heat of melting of a completely crystalline PE [27].

(1) $$x=\frac{\Delta H_{i}}{\Delta H_{0}}\times 100.$$

#### 2.2.4. Fourier-Transform Infrared Spectroscopy (FTIR)

The analysis of chemical composition was performed by Fourier-transform infrared spectroscopy using an FTIR spectrometer Spectrum 100 (Perkin Elmer, Beaconsfield, United Kindom) at a temperature of (22 ± 2) °C in the wavenumber range of 4600 ≤ n ≤ 650 cm^−1^ in the transition light and by an attenuation total reflection method (ATR). The changes in the chemical composition of the materials after exposure in the aqueous and soil media, as well as after fungi biodeterioration were studied.

#### 2.2.5. Kinetics of Water Absorption

The water absorption degree of the films was determined into distilled water at (30 ± 2) °C for 45 days according to DIN EN ISO 62:2008-05. The method consists of determining the change in sample mass and appearance after exposure in the model medium (distilled water) at the stated intervals. The measurements were carried out for 45 days until an equilibrium water absorption. Additionally, changes in the PE crystalline structure and the degree of extractable substances were determined.

#### 2.2.6. Mycological Test with Mold Fungi

The ability of materials to support the fungal growth was evaluated using different cultures of fungi according to ISO EN 846:1997. Mold fungi strains from the collection of the Department of Mycology and Algology (Lomonosov Moscow State University) were used: I—*Trichoderma harzianum Rifai*; II—*Penicillium chrysogenum Thorn*; III—*Fiisarhim moniliforme Sheld*; IV—*Chaetommm glohosimi Kunze*; V—*Trichoderma asperellum Samuels Lieckf and Nireberg*.

Two series of experiments were carried out: with potato nutrient culture medium as a substrate for fungi cultivation and with a saturated solution of potassium chloride (samples were used as a sole source of nutrition). Incubation of the samples (size of 1 × 1 cm) inoculated by the fungi spore suspension or by dry process (5 × 10^4^ spores/mL) was carried out at a relative humidity greater than 90% at (28 ± 2) °C using a moist chamber for 25 days with an intermediate inspection after 4 and 14 days.

The morphology and intensity of fungal growth were evaluated by an optical microscope according to ISO EN 846:1997. The criteria of biofouling assessment used in this work are shown in Table 3.

After the exposure for 25 days, the biomass gain and the fungal intensity of growth were assessed by optical microscopy [28]. The values of hyphae length and spores’ quantity were measured.

The average length value of hyphae (cm) was calculated by an Equation (2), where *a*—value of hyphae length in one microscopic field (units of ocular scale); *b* = 9.091 µm—unit value of ocular scale (μm); *S_1_* = 108 μm^2^—specimen area; *S_2_* = 2.34 × 10^7^ μm^2^—area of the microscopic field.

(2) $$L=a\times b\times \frac{S_{1}}{S_{2}}\times 10^{-4}$$

The biomass value (fungal mycelium weight) was calculated using Equation (3), where *L*—average length value of hyphae calculated according to Equation (2); *r* = 2.5 × 10^−4^ cm—value of hyphae radius; *ρ* = 1.05 g·cm^−3^—weight density of mycelium.

(3) $$P=L\times \varrho \times \pi \times r^{2}.$$

#### 2.2.7. Biodegradability Test in Soil Medium

The biodegradation test was carried out by exposure of the film samples into the soil for 45 and 90 days. The biodegradation test on the recovered soil was carried out in accordance with ASTM D 5899 to investigate the biodegradability of the material. The soil was made of sand, garden soil, and horse manure taken in equal amounts. The resulting soil was held for two months at (20 ± 5) °C with daily stirring and humidity maintained at (60 ± 10)%. The film samples were immersed vertically in the prepared soil and exposure at (22 ± 3) °C and relative humidity of (60 ± 10)% for 45 and 90 days. The change in appearance, weight and chemical composition (by FTIR) of the materials were investigated after exposure in the soil medium. Three specimens of each composite were tested.

The work was carried out using scientific equipment of the Center of Shared Usage «New Materials and Technologies of Emanuel Institute of Biochemical Physics» and Joint Research Center of Plekhanov Russian University of Economics.

## 3. Results

### 3.1. Structure and Properties

**Figure 1:**
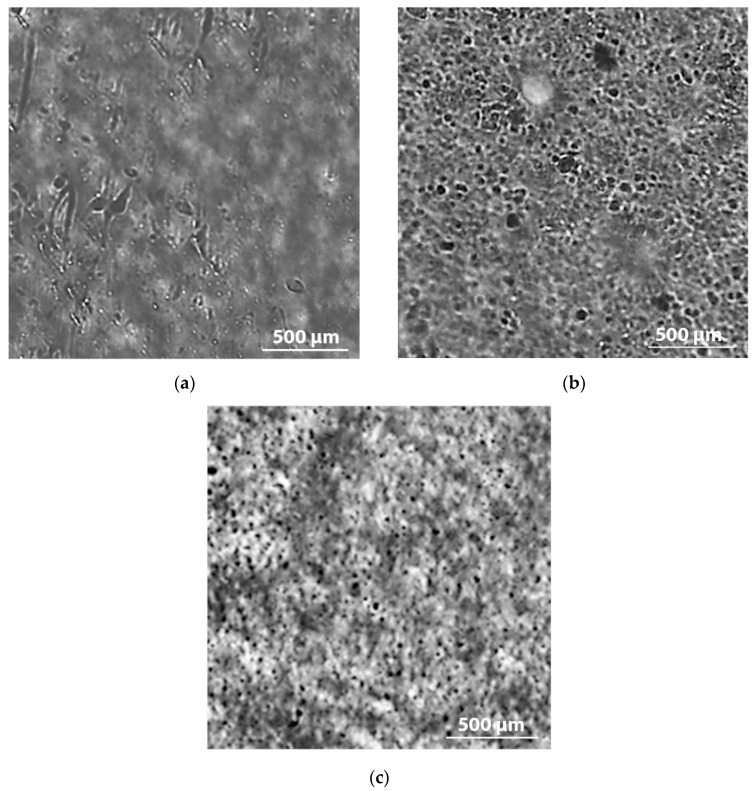
The microphotographs of the PE/NR composite samples containing 10 (**a**), 20 (**b**) and 30 (**c**) wt% of NR (transmitted light, at a magnification of 200×).

The addition of filler to the polymeric matrix leads to significant changes in the morphology of the composite material and the macromolecular mobility of the boundary layers. By means of optical microscopy, the microstructure of composite materials was analyzed. Figure 1 shows the selected microphotographs of the PE/NR composites. When compounding PE with NR, the formed system represented a PE matrix with domains of NR dispersed inside. Having an elastomeric nature, NR behaved as a flexible dispersed filler in the PE matrix. In this case, PE/NR systems cannot be considered as a blend of two thermoplastic polymers. The rubber domains in the composites were characterized by a size of 10–100 μm. It was found that with an increase in the content of NR in the PE matrix, more uniform dispersion of domains occurred with a decrease in their sizes (Figure 1).

**Figure 2:**
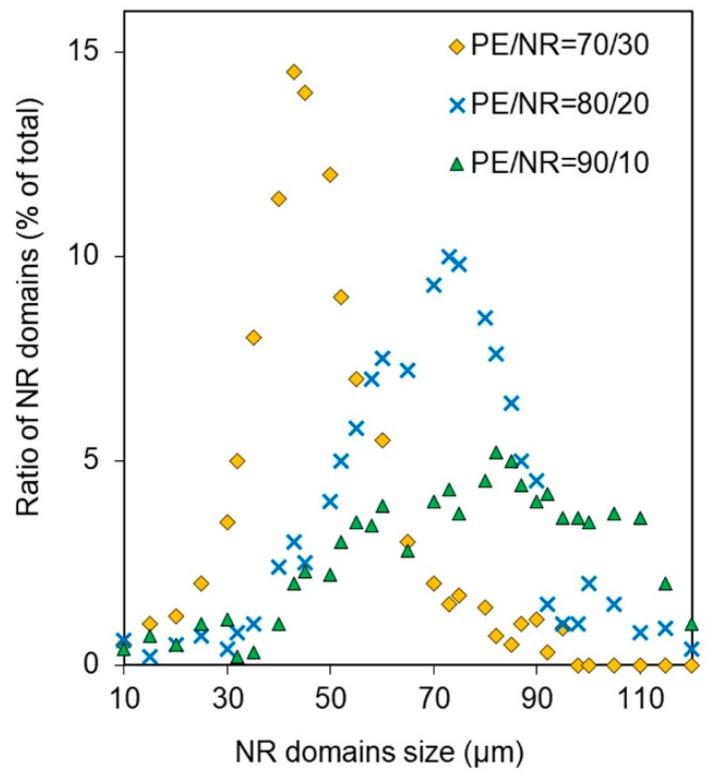
The size distribution of NR domains in the PE/NR composites containing 10, 20 and 30 wt% of NR.

The size distribution of NR domains in the PE matrix is presented in Figure 2. According to the obtained results, the composites PE/NR = 70/30 had a more uniform distribution of rubber particles with an average size of (45 ± 5) µm. The composites with 20 wt% of NR in the PE matrix had a wider distribution throughout domains sizes. In the case of small NR additives, an inhomogeneous structure with sufficiently large inclusions of the NR phase was observed in PE/NR composites.

**Figure 3:**
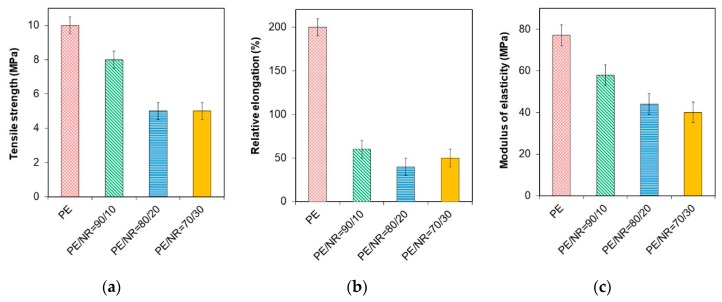
The parameters of tensile strength at break (**a**), relative elongation at break (**b**), modulus of elasticity (**c**) under tension for neat PE and the PE/NR composites with NR content of 10, 20, 30 wt%.

The microstructure of the PE/NR composites determines their behavior under tension. Figure 3 demonstrates the basic parameters of the mechanical properties of the investigated composite films. Adding NR to PE contributes to reducing the relative elongation at break. When 10 wt% of NR was added to PE, the value of relative elongation at break reduced to four-fold the relative elongation of neat PE. As the content of NR increased, no further changes were observed. It was defined that the tensile strength and Young’s modulus for the PE/NR composites were about two times lower than these values for neat PE.

Using DSC analysis, the melting and crystallization behavior of PE as a part of the composites was studied. The analysis was conducted for the initial samples, as well as the samples after aging in the aqueous and soil media. The resulting parameters are presented in Table 4. According to DSC results, as the content of NR increased, the melting peak of PE shifted in the region of lower temperatures. The exposure to water had an insignificant influence on the PE component of the composites. The degree of PE crystallinity increased from 29% to 34% after exposure to the soil medium for 90 days.

### 3.2. Degradation Study

**Figure 4:**
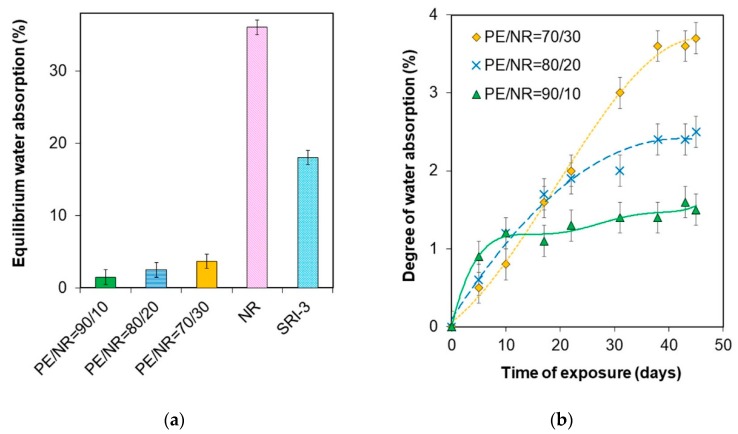
(**a**) The equilibrium degree of water absorption of PE/NR composites, NR and SRI-3 after 45 days in the aqueous medium; (**b**) The water absorption curves for the PE/NR composites with NR content of 30 (1), 20 (2), 10 (3) wt%.

Figure 4 shows the data of equilibrium water absorption and the water absorption curves for the composites under investigation. NR was characterized by a high degree of water absorption (36 ± 0.2)%, which made it susceptible to soil microorganisms. For synthetic poly(isoprene) rubber SKI-3, the degree of equilibrium water absorption was lower (18 ± 0.2)% than for NR. This fact could be related to the difference in the structure and chemical composition of the materials. Natural rubber consists almost entirely of the cis-1,4-polyisoprene with different accessory substances. SKI-3 contains only trans-1,4-polyisoprene. According to the data, the values of equilibrium water absorption for the PE/NR composites increased with decreasing PE content. The water absorption for the composite PE/NR = 70/30 was 3.7 wt% after 45 days in the aqueous medium.

**Figure 5:**
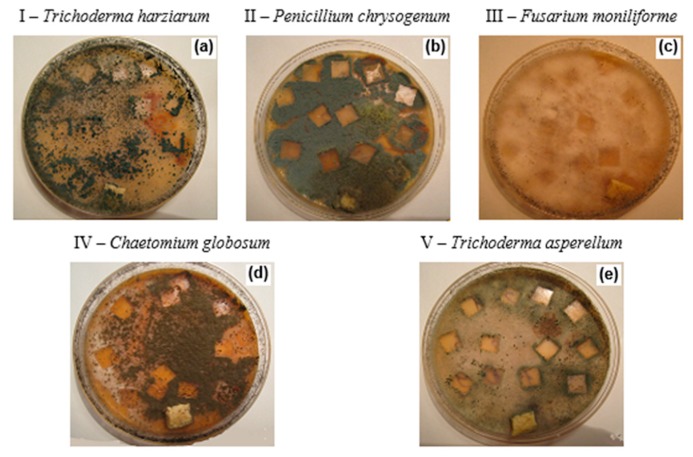
The photographs of composite samples of PE/NR = 70/30 after 25 days from the inoculation by mold fungi spores of *Trichoderma harzianum Rifai* (**a**), *Penicillium chrysogenum Thorn* (**b**), *Fiisarhim moniliforme Sheld* (**c**), *Chaetommm glohosimi Kunze* (**d**), *Trichoderma asperellum Samuels Lieckf* and *Nireberg* (**e**) (potato nutrient culture medium).

Using the fungal test, two parameters were estimated: the intensity of samples biofouling in dynamics and the biomass value after 25 days of incubation. By way of an example, the photographs of a composite sample based on PE/NR = 70/30 after 25 days from inoculation by fungal spores (potato nutrient culture medium) are presented (Figure 5).

**Figure 6:**
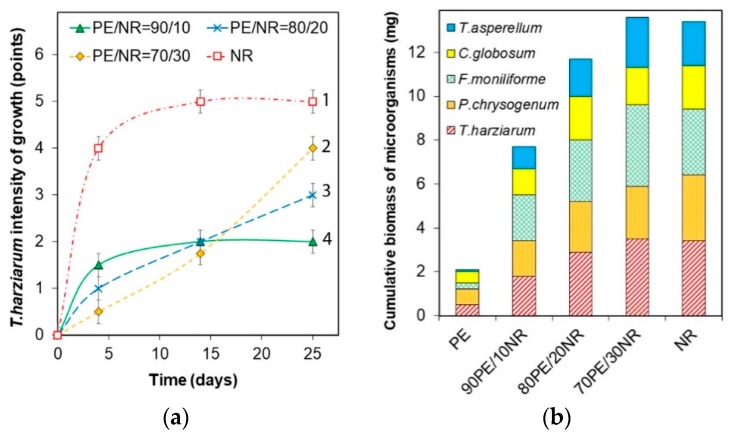
(**a**) The intensity of biofouling of NR and PE/NR composites by a test culture *Trichoderma harzianum* (potassium chloride medium); (**b**) The cumulative biomass gain value (Δ ± 0.1 mg) after 25 days from the inoculation of NR and PE/NR composites by fungal spores (potassium chloride medium).

It was determined that most fungal stains had the common tendencies of growth depending on NR content in the composites. As an example, the samples biofouling assessment according to five-scoring scale (ISO EN 846:1997) for a test culture of *T. harzianum* is shown in Figure 6a. The composite with 30 wt% of NR was characterized by a higher rate of growth compared to the composites with a lower content of NR. The cumulative biomass gain after 25 days from inoculation with fungal spores allowed to determine the hybrid composite of PE/NR = 70/30 as the most biodegradable among the investigated composites (Figure 6b). Moreover, neat NR and PE composite with 30 wt% of NR has a similar cumulative biomass gain values.

The biodegradability of the PE/NR composites under environmental conditions was investigated by a full-scale soil test. Preliminarily neat natural rubber was tested under the soil medium. The reduction in weight of neat NR samples after exposure for 90 days in soil was 38.3% that indicated a high biodegradability of natural rubber (Table 5). The mass loss of PE/NR = 70/30 composite after 90 days in soil was about 7%.

After exposure of the composites to both aqueous and soil media, the appearance, structure and chemical composition changes of the materials were analyzed. The degree of crystallinity and the range of crystalline particles of PE increased after both kinds of treatment (Table 5). The higher the content of NR in the composites was, the more intense structural changes by aqueous and soil media occurred.

Table 5 shows the results of the evaluation of different absorbance ratios from FTIR for analyzing the changes in the chemical composition of the materials after a soil test. The peak at 1463 cm^−1^ corresponds to the bending deformation vibrations of the CH_2_–group in polyethylene. The peaks at 836 cm^−1^ and 1368 cm^−1^ are characteristic for C–H bending vibrations in =CH and –CH_3_ groups of cis-1,4-polyisoprene, correspondently [29]. Based on the absorbance ratios of the bands at 836 and 1463 cm^−1^ (D_836_/D_1463_) and the bands at 1376 and 1463 cm^−1^ (D_1376_/D_1463_), the quantitate analysis of changing NR content in the materials compared to PE was conducted. According to the results, the changes in chemical composition were revealed for the PE/NR composites containing 20 and 30 wt% of NR exposed to the soil medium. The intensity of bands at 838 cm^−1^ and 1376 cm^−1^, which are specific for NR, decreased by 38% and 29%, correspondently, for the composite with 30 wt% of natural rubber after 90 days in the soil medium. The observed loss in NR content by FTIR (ATR-method) was higher than the mass loss of the PE/NR composites due to the higher rate of degradation for the surface layers in comparison with the internal layer.

**Figure 7:**
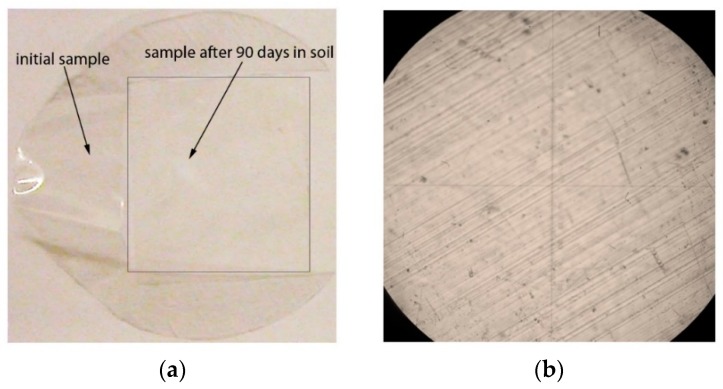
(**a**) The appearance changes of the films based on neat PE (initial sample and sample after 90 days in the soil medium); (**b**) The microphotograph of the neat PE after exposure in the soil medium for 90 days (transmitted light, at a magnification of 100×).

After 45 and 90-day soil tests, visual inspection and analysis by optical microscopy were conducted. There were no changes in the appearance of the samples of neat PE after exposure in the soil medium. Figure 7a shows photographs of the initials sample of neat PE and the sample of PE after 90 days in the soil medium. Figure 7b demonstrates the microphotograph of neat PE exposed for 90 days in soil. No defects, staining and biological fouling were observed on the polyethylene samples.

**Figure 8:**
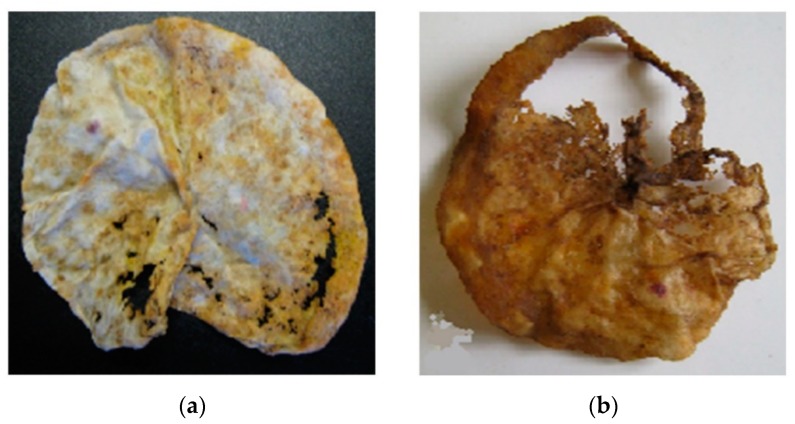
The appearance changes of the films based on neat NR after 45 (**a**) and 90 (**b**) days in the soil medium.

**Figure 9:**
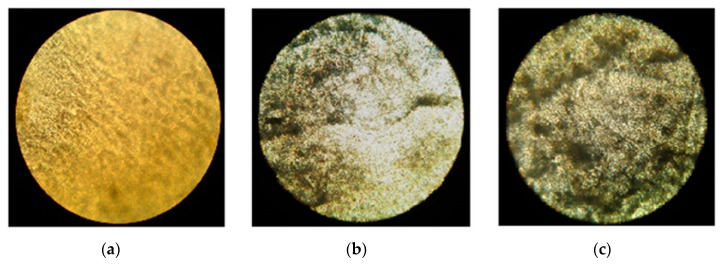
The microphotographs of the composite of PE/NR = 70/30: initial sample (**a**), sample after exposure in the soil for 45 days (**b**), sample after exposure in the soil for 90 days (**c**) (transmitted light, at a magnification of 100×).

The obtained photographs and microphotographs of NR and the PE/NR composites are presented in Figure 8 and Figure 9, respectivley. Figure 8 demonstrates appearance changes in the films based on neat NR after 45 and 90 days in soil medium. Numerous defects, color darkening and deterioration by soil microorganisms of NR samples are visible by the naked eye. 

For the PE/NR composites, a loss of transparency and appearance of colored spots after the soil test were detected by the naked eye. By virtue of optical microscopy, biofouling of the PE/NR composites by mold fungi with mycelium growth was revealed. The microphotographs of the composite based on PE/NR = 70/30 are shown in Figure 9. The intensity of biofouling of the PE/NR = 70/30 sample after a 90-day soil test was 4 points (according to ISO EN 846:1997). More than 50% of this sample surface was covered with microorganisms’ growth that indicates the material contained enough nutritive components enabling for the growth of soil microorganisms.

**Figure 10:**
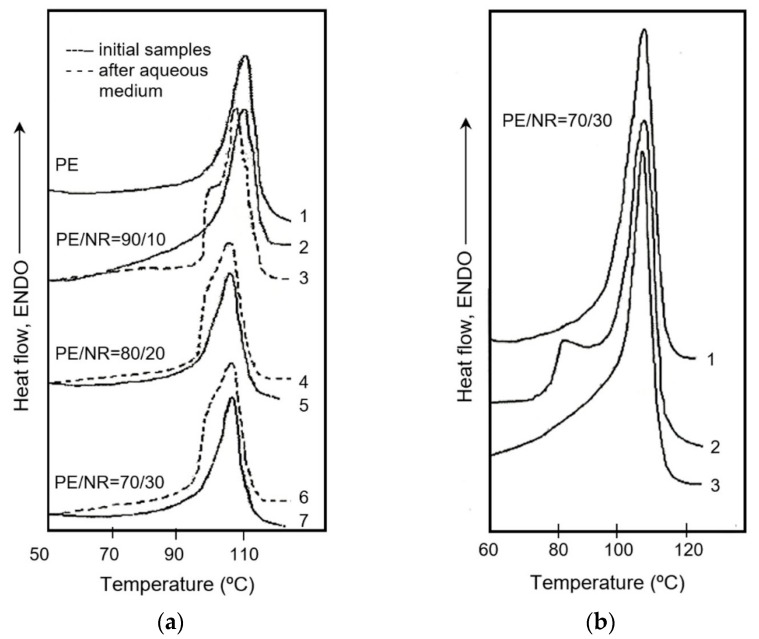
The DSC melting curves: (**a**) PE (1) and PE/NR composites with NR content of 10 (2,3), 20 (4,5), 30 (6,7) wt%: solid lines 1, 2, 5, 7—initial samples; dashed lines 3, 5, 7—samples after exposure in the aqueous medium for 45 days; (**b**) PE/NR = 70/30 composite: 1—initial sample; 2—sample after exposure in soil for 90 days (first heating); 3—sample after exposure in soil for 90 days (second heating).

Figure 10 shows the DSC curves of the melting of the initial samples, as well as the samples exposed to the aqueous and soil media. There were no changes in the crystalline structure of neat PE. In contrast, the crystalline structure of PE in the composites with NR was changed under the influence of the aqueous and soil media, which was apparent in the bimodality of the PE melting peak. An additional low temperature melting peak of PE was observed for the PE/NR = 70/30 composite samples exposed in the moist soil for 90 days (Figure 10b, curve 2). At the second melting of this PE/NR composite, the PE endothermic peak on the DSC curve becomes monomodal (Figure 10b, curve 3).

## 4. Discussion

The phase structure of the PE/NR composites including the character of distribution of NR domains has a significant effect on performance properties and mechanism of degradation of the composite materials. It was determined that the composites with NR content of 30 wt% were characterized by a smaller and narrower size distribution than those with a lower NR content. Decreasing in NR domain size could be evidence of forming an abnormal overstrained system with excess free energy. According to the literature, such polymeric systems are highly susceptible to destructive factors [30]. In addition, a more uniform distribution of NR domains in the PE matrix for the composite of PE/NR = 70/30 indicates better interaction of components in the melt.

The entirely new structure of composite material is responsible for new mechanical properties. The addition of natural filler to the polymer matrix provides defective zones at the interface region between the polymer and filler particles [31]. As a result, the plastic yield under the tension of the filled composites decreased significantly. Nevertheless, PE/NR composites under investigation are characterized by satisfying elastic properties compared to another composite based on polyethylene with dispersed fillers. The good mechanical properties of the PE/NR composites could be caused by high elasticity and uniform distribution of rubber particles. In contrast, in case of adding 10–30 wt% of different dispersed fillers to polyethylene (cellulose, flax straw, wood flour, etc.), the elongation at break value decreased by 90 ± 5%, which indicated the brittle fracture of materials [14,15,16,17]. The samples were fractured in the yield region before the plastic flow.

On the other hand, NR domains do not reinforce the PE matrix as opposed to most of the cellulosic fillers. In the case of addition 10 wt% of NR to PE, the tensile strength and modulus of elasticity went down by (20 ± 5)% compared to neat PE. Provided that NR content in the polyethylene matrix increases to 20 wt%, the further reduction occurred. The content of 20 wt% of NR in the composite is critical. In the case of the further increasing NR content, the tensile strength of composites stopped changing.

According to the DSC analysis, as the content of NR increased, PE melting onset temperature decreased. Apparently, the presence of NR prevents PE crystallization making the perfection of the crystallines lower. The degree of crystallinity of PE changed slightly. The range of PE crystalline particles was determined by width at height of melting peaks. When NR content varied, this value changed slightly.

The required mechanical and thermal properties of the PE/NR composites determined the availability of developing such composites for use in different consumption fields. Nevertheless, the main feature of the materials under investigation is biodegradability under environmental conditions. Therefore, a significant part of this study was devoted to this aspect. The biodegradability of neat NR and the composites based on PE and NR was studied by a water absorption test, a microbiological test with mold fungi and a full-scale soil test.

Water is widely distributed in the environment and is able to penetrate through the surface layers of material to diffuse deeper, causing both plasticizing and wedging action (Rebinder effect) [32]. Besides that, the bioavailability of NR in the PE matrix appears to depend on the polymeric matrix structure influencing the value of water absorption. Investigation of water absorption and kinetics of this process allows analyzing structural features and predicting changes while the biodegradation process of the composites.

NR was characterized by a high degree of water absorption (36 ± 0.2)%, which made it susceptible to soil microorganisms. For synthetic poly(isoprene) rubber SKI-3, the degree of equilibrium water absorption was lower (18 ± 0.2)%. This fact could be related to the difference in rubbers structure (SKI-3 consists of poly(trans-isoprene)) and accessory substances in NR [33].

According to the data of the water absorption test, the PE/NR composites had a higher water absorption degree compared to neat PE. Besides, the equilibrium water absorption for the composites increased with decreasing in PE content. In accordance with the kinetics of water absorption for the PE/NR composites, the rate of water absorption decreased in proportion to increasing NR content, which could be caused by high dispersion of NR domains in the PE matrix with increasing of the phases contact area.

The enhanced degree of water absorption facilitates penetrating vital activity products of microorganisms (acids and enzymes) into the materials that lead to hydrolysis of NR [34]. The products of NR hydrolysis, having lower molecular weight and higher diffusion coefficient through the polymer matrix, are able to leave the samples that in turn is resulting in the weight decrease of materials at exposure in soil. Moreover, PE in the PE/NR composites is exposed to the impact the absorbed water.

The analysis of materials bioresistance to mold fungi is one of the common model experiments for determining biodegradability of materials particularly polymeric composites. It was determined that fungal cultures of *T. harzianum* and *F. moniliforme* had the most intensive spreading on the PE/NR composites. So, these cultures could be indicated as the main biodestructors of PE/NR composites. The color change of the test samples was observed for cultures of *P. chrysogenum* and *C. globosum* that could be related to metabolites released by microorganisms (including pigments).

According to the results, most fungal stains had the common tendencies of growth depending on NR content in the composites. The composites with 30 wt% of NR were characterized by a slower growth rate compared to the composites with a lower content of NR. Apparently, this fact is connected with the accessibility of NR domains in the PE matrix. Since the composite of PE/NR = 90/10 contained large-dimension particles of NR (about 90 µm), NR domains located close to the sample’s surface could be easily assimilated by fungi and used as a nutrient source. However, the remaining NR domains encapsulated in the PE matrix are not accessible to microorganisms. On the contrary, NR in the composites of PE/NR = 70/30 were fine-dispersed particles that could be a limiting factor of fungal growth at the first period of cultivation.

The biodegradability of PE/NR composites under environmental conditions was investigated by a full-scale soil test. By virtue of optical microscopy, biofouling of the composites by mold fungi was revealed. The release of microorganisms’ metabolites, including melatonin type pigments, apparently leads to discoloration of the materials.

The mass loss is a basic criterion of the biodegradation rate under environmental conditions. The micromycete growth and the intensity of materials destruction evaluated by a weight loss after the soil tests largely depended on the NR content. Therefore, the samples containing 30 wt% of NR had the greatest weight loss and a significant change in the appearance in comparison with the other samples. The intensive weight reduction of these materials exposed in the soil is induced by a high biodegradability of materials in the environment. For composite samples with 10 and 20 wt% of NR, the weight reduction occurred during the first 1.5 months, after that the weight loss values remained unchanged. Probably, for these compositions, NR is encapsulated in the PE matrix; hence, microorganisms could assimilate only the surface NR particles. According to the results of a 90-day soil test, a mass loss of the PE/NR = 70/30 composite was about 7%.

After exposure to composites in both the aqueous and soil media, the structural changes of PE and NR were observed. It was determined that water including soil moisture promoted the recrystallization of PE crystallines. An increase in the degree of crystallinity of PE could be related to an enhanced degree of water absorption and an effect of NR particles on the material’s structure. The phenomenon of PE recrystallization in the aqueous medium is connected with relaxation and the formation of a more ordered crystalline structure under the plasticizing effect of water. The structural reorganization of PE crystallines after exposure in water for 18 months was described in the work [35]. After exposure to the PE/NR composites in the moist soil, an additional peak was observed in the DSC curves at the melting of the exposed samples. This additional endothermic peak is probably responsible for the presence of bound moisture in these materials.

In addition, the changes in chemical composition were revealed for the PE/NR composites containing 20 and 30 wt% of NR exposed to the soil test. The decrease of IR absorption bands intensity of 836 cm^−1^ (C–H bending vibrations of sp^2^ hybridized carbons in C(CH_3_)=CH groups) and 1368 cm^−1^ (C–H bending vibrations in CH_3_–groups) gave evidence of the utilization of NR by soil microorganisms. Like that, the rate of NR biodegradation was higher for composites with a higher content of NR.

The biodegradation of the developed composites could be described as a complex process including several stages. It is known that bioassimilation and subsequent degradation of polymeric materials start from adhesion and attachment of fungal spores to the substrate [36,37]. The work results demonstrated a high ability of the composites to biofoul by mold fungi that confirms susceptibility of the PE/NR composites to biodegradation by microorganisms.

After the biofouling of the sample’s surface occurs, availability and usability as a nutrient source of the substrate play important roles at the further stage of biodegradation [38]. The usability of the filled composites by microorganisms is mainly dependent on the filler biodegradability, the diffusing properties of the polymeric matrix, the composite structure including the interfacial area between phases, as well as the degree of water absorption. Under the action of microorganism’ enzymes and soil moisture on the composite materials, hydrolysis of the substances contained in the composites can occur [33]. According to the work results, natural rubber had an enhanced biodegradability (the period of full degradation of NR is about half of a year). Moreover, the PE/NR composites under investigation were characterized by an increased degree of water absorption that facilitates the penetration of vital products of microorganisms (acids and enzymes) into the materials, which leads to hydrolysis and oxidation [38,39]. In turn, the decomposition products having a low molecular weight and a higher diffusion coefficient can leave the samples, which leads to a decrease in the weight of materials when exposed in the soil.

It was revealed that the composites were characterized by an intensive weight reduction at the first three months in the soil medium (the mass loss of the PE/NR = 70/30 composite was about 7%). Provided that the FTIR analysis, this mass loss was related to the bioassimilation of NR domains by soil microorganisms. This fact is often observed for such types of biodegradable polymeric composites [14,16]. The further degradation is not limited by the natural rubber bioassimilation. Because of the NR utilization in the matrix of PE, the composites structure became porous with a great deal of different defects. For this reason, the uniform distribution of biodegradable filler (in this case, natural rubber) in the polymeric matrix is important for the degradation process. Moreover, the intensive growth of microbiota on the materials is accompanied by the formation of microorganisms’ metabolites and the occurrence of mechanical stresses, which are the causes of destruction. In several papers [40,41,42], it was shown the biochemical destruction of poly(ethylene) under the action of certain enzymes of micromycetes and bacteria accompanied by a decrease in the molecular weight of PE. 

Therefore, the main purpose of introducing natural additives into poly(olefins) is enhancing their bioavailability and biofouling, due to natural fillers are easier than poly(olefins) to be exposed to physicochemical and biological environmental factors. After the microorganisms have attached to the surface of the polymer material, biofouling and degradation of the material occur under the action of intracellular enzymes of microorganisms, which leads to the polymer depolymerization. Ultimately, the fragments of polyethylene macromolecules become short enough for assimilation by microorganisms and mineralization. According to the previous studies [43], microorganisms are able to assimilate paraffins with a molecular weight of less than 600.

The intensity of poly(ethylene) degradation in such types of composite materials is commonly evaluated after a longer period of degradation (1–2 years). The mass loss, the accumulation of oxidation products, and the decrease in the degree of crystallinity are used as criteria for assessing the destruction of polyethylene [16,44]. The further study of the degradation process of the developed composites based on poly(ethylene), natural rubber and technological additives is planned.

## 5. Conclusions

The development of composites based on poly(ethylene) and natural rubber allows modifying polyethylene structure and properties including biodegradability. Adding natural rubber makes polyethylene more susceptible to degradation agents, including moisture, corrosive chemical substances, oxidizing agents, and products of soil microorganisms’ metabolism. As a result, the composites of polyethylene with natural rubber has a higher level of biodegradability under environmental conditions compared to neat polyethylene. In accordance with the obtained results, the biodegradability of polyethylene/natural rubber composites was revealed by the changes in materials weight, appearance and physical properties, poly(ethylene) structure as well as chemical composition. In addition, the composites based on polyethylene with natural rubber additives have satisfactory mechanical and technological properties that determine the suitability of such materials for application as packaging and agricultural films with advanced biodegradability.

## Figures and Tables

**Table 1:** Main characteristics of low-density poly(ethylene) (PE) [2,15].

Parameter	Method	Characterization
Molecular mass characteristics	Gel filtration chromatography, at 140 °C, 1,2,4-trichlorobenzene as a solvent	M_w_ = 1.0 × 10^5^, M_n_ = 1.5 × 10^4^, M_w_/M_n_ = 7.03
MFI (melt flow index)	Capillary viscometry, at 190 °C and load of 2.16 kg	1.6 ± 0.1 g·10 min^−1^
Density	Hydrostatic weighing, in ethanol 95 vol%	0.923 ± 0.001 g·sm^−3^

**Table 2:** Chemical composition of natural rubber (NR), grade SVR 3L [27].

Substance	Content, wt%
Poly(cis-1,4-isoprene)	91–96
Protein and amino acids	2–3
Resins	2–3
Soot indicator	0.5
Volatiles	0.8
Transition metal compounds	<1
Other	<1

**Table 3:** Assessment of microorganisms’ growth (ISO EN 846:1997).

Intensity of Growth (Points)	Evaluation
0	No growth was seen under the microscope. The material does not contain any nutritive component.
1	No growth was seen by the naked eye but was visible under the microscope.
2	Growth was visible to the naked eye. 25% of the test sample surface was covered with microorganisms growth. The material contains nutritive components providing a slight growth of microorganisms.
3	Growth was visible to the naked eye. 50% of the test sample surface was covered with microorganisms growth.
4	Growth was visible to the naked eye. More than 50% of the test sample surface was covered with microorganisms growth. The material contains enough nutritive components enabling the growth of microorganisms.
5	Heavy growth covered the entire surface of the test sample.

**Table 4:** The thermophysical parameters of PE (melting point (*T_m_*), degree of crystallinity (χ), peak width at half height (Δ*T*½)) for initial samples and samples after aging in the aqueous and soil media.

NR, wt%	Initial Samples			Samples after Exposure in Aqueous Medium for 14 Days			Samples after Exposure in Soil Mediumfor 90 Days	
	*T_m_*, °C (Δ ± 0.2 °C)	χ, % (Δ ± 1%)	Δ*T*½, °C (Δ ± 0.2 °C)	*T_m_*, °C( Δ ± 0.2 °C)	χ, %(Δ ± 1%)	Δ*T*½, °C (Δ ± 0.2 °C)	*T_m_*, °C (Δ ± 0.2 °C)	χ, % (Δ ± 1%)
0	110.0	29	10.6	110.0	29	10.5	110.0	29
10	109.5	29	10.4	106.5	30	11.5	108.0	29
20	105.5	29	10.0	105.5	32	13.0	108.0	32
30	106.0	29	9.8	106.0	31	12.0	107.5	34

**Table 5:** The changes in composite materials after exposure in the soil medium.

NR, wt%	Mass Loss, % (after Exposure in Soil for 45 Days)	Mass Loss, % (after Exposure in Soil for 90 Days)	D_836_/D_1463_* Initial Samples)	D_836_/D_1463_ (after Exposure in Soil for 90 Days)	D_1376_/D_1463_** (Initial Samples)	D_1376_/D_1463_ (after Exposure in Soil for 90 Days)
0	0	0	0	0	0	0
10	1.3	1.3	–	–	–	–
20	1.5	1.5	0.0920	0.0850	0.2310	0.2180
30	2.7	7.2	0.1668	0.1029	0.3409	0.2448
100	16.2	38.3	–	–	–	–

* D_836_/D_1463_—The absorbance ratio of the bands at 836 and 1463 cm^−1^; ** D_1376_/D_1463_—The absorbance ratio of the bands at 1376 and 1463 cm^−1^.
